# Prediction of the Cause of Fundus-Obscuring Vitreous Hemorrhage Using Machine Learning

**DOI:** 10.3390/diagnostics15030371

**Published:** 2025-02-04

**Authors:** Jinsoo Kim, Bo Sook Han, Joo Eun Ha, Min Seon Park, Soonil Kwon, Bum-Joo Cho

**Affiliations:** 1 Department of Ophthalmology, Hallym University Sacred Heart Hospital, Hallym University College of Medicine, Anyang 14068, Republic of Korea; 2 Medical Artificial Intelligence Center, Hallym University Medical Center, Anyang 14068, Republic of Korea

**Keywords:** vitreous hemorrhage, fundus-obscuring vitreous hemorrhage, etiology, machine learning, artificial neural network

## Abstract

**Objectives:** This study aimed to predict the unknown etiology of fundus-obscuring vitreous hemorrhage (FOVH) based on preoperative conditions using machine learning (ML) and to identify key preoperative factors. **Methods:** Medical records of 223 eyes from 204 patients who underwent vitrectomy for FOVH of unknown etiology between January 2012 and July 2022 were retrospectively reviewed. Preoperative data, including demographic information, systemic disease, ophthalmic history, and retinal status of the unaffected eye, were collected. The postoperatively identified etiologies of FOVH were categorized into six groups: proliferative diabetic retinopathy (PDR), retinal vein occlusion (RVO) or rupture of retinal arterial macroaneurysm, neovascular age-related macular degeneration (nAMD), retinal tear, Terson syndrome, and other causes. Four ML algorithms were trained and evaluated using seven-fold cross-validation. **Results**: The ML algorithms achieved mean accuracies of 76.2% for artificial neural network, 74.5% for XG-Boost, 74.4% for LASSO logistic regression, and 68.5% for decision tree. Key predictive factors commonly selected by the ML algorithms included PDR in the fellow eye, underlying diabetes mellitus, subarachnoid hemorrhage, and a history of retinal tear, RVO, or nAMD in the affected eye. **Conclusions**: The unknown etiology of FOVH could be predicted preoperatively with considerable accuracy by ML algorithms. Previous ophthalmic conditions in the affected eye and the condition of the fellow eye were important variables for prediction. This approach might assist in determining appropriate treatment plans.

## 1. Introduction

Vitreous hemorrhage (VH), characterized by the presence of blood within the vitreous cavity, is a common cause of acute or subacute vision loss [1], with a reported incidence of 7 to 48 cases per 100,000 people annually [2,3]. The pathophysiology of VH involves the rupture of normal retinal vessels due to mechanical forces, bleeding from vulnerable new retinal vessels, or the extension of hemorrhage from other sources within the eye [1,4]. Spontaneous VH can result from various vitreoretinal diseases, such as proliferative diabetic retinopathy (PDR), retinal vein occlusion (RVO), retinal tears, or neovascular age-related macular degeneration (nAMD) [4].

Fundus-obscuring vitreous hemorrhage (FOVH), which obscures the entire vitreous cavity and precludes fundus visualization, poses significant challenges for clinicians in determining appropriate treatment strategies [5,6]. The difficulty lies in identifying the underlying cause of VH. Clinicians are faced with the decision between non-surgical management, closely monitoring the patient for possible RVO or PDR, and early vitrectomy in anticipation of retinal tears that may lead to retinal detachment (RD) [6]. Thus far, very limited research has been conducted on predicting the etiology of FOVH preoperatively.

Recently, the application of artificial intelligence and machine learning (ML) techniques has enabled various predictions in the field of ophthalmology by utilizing relevant data [7,8]. ML algorithms offer many non-linear predictive methods that traditional linear models cannot provide and might enable the prediction of the underlying cause of FOVH. Therefore, the present study aimed to predict the unknown etiology of FOVH using preoperative information only via ML algorithms. To the best of our knowledge, this is the first study to predict the cause of VH without direct visualization of the fundus status.

The contributions of this study could be, first, demonstrating that various etiologies can be predicted using preoperative variables, and second, identifying key factors for assessing the causes of FOVH, such as the presence of PDR in the fellow eyes. Additionally, this study highlights that such analyses are feasible through ML approaches.

The structure of this paper is as follows: Section 2 details the methodology employed in this study, including patient enrollment criteria, data collection processes, and the ML algorithms utilized for prediction. Section 3 provides a comprehensive overview of the results, emphasizing the predictive performance of different ML models. Section 4 discusses the implications of these findings, highlighting their relevance in clinical practice.

## 2. Materials and Methods

### 2.1. Subject Enrollment

**Figure 1:**
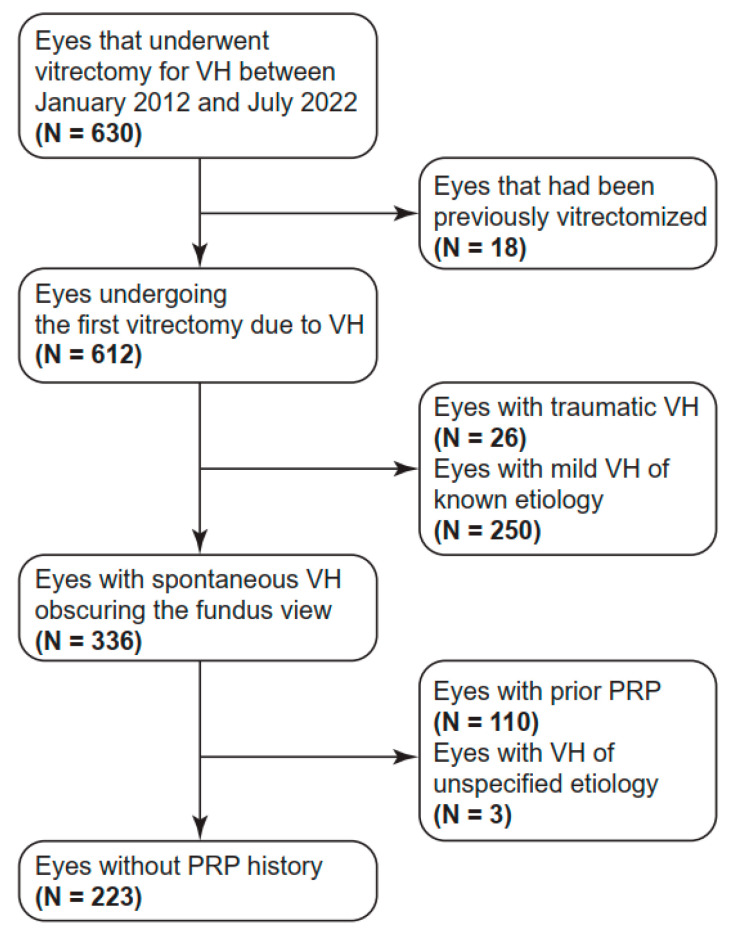
Enrollment process of patients with fundus-obscuring vitreous hemorrhage. PRP, pan-retinal photocoagulation; and VH, vitreous hemorrhage.

The present study consecutively enrolled patients who underwent their first pars plana vitrectomy (PPV) at Hallym University Sacred Heart Hospital in Korea between January 2012 and July 2022 for FOVH of unknown etiology. The enrollment process is illustrated in Figure 1. Patients younger than 19 years old at the time of operation or those with traumatic VH or mild VH that allowed for identification of the cause of VH were excluded. Eyes with a history of pan-retinal photocoagulation (PRP) or PPV were also excluded.

The subjects received PPV early or late, depending on the operator’s decision. In cases of suspected retinal tear or RD, immediate vitrectomy was performed; otherwise, patients were followed for 1 to 2 months, and surgery was performed if VH did not resolve. This study was approved by the institutional review board of Hallym University Sacred Heart Hospital (IRB number, HALLYM 2022-09-007) and was conducted in accordance with the Declaration of Helsinki. Informed consent requirements were waived due to the retrospective nature of this study and the use of anonymized patient data.

### 2.2. Data Collection

The medical records of the subjects were retrospectively reviewed, and various preoperative conditions were investigated for patient demographics, systemic medical conditions, ophthalmic history of the affected eye, and current retinal status and ophthalmic history of the fellow eye. Demographic data included age at the operation and sex. Data related to systemic medical conditions involved the preoperative presence of underlying systemic diseases, including diabetes mellitus (DM), hypertension (HTN), cerebrovascular accident (CVA), chronic kidney disease (CKD), coronary artery disease (CAD), and subarachnoid hemorrhage (SAH). The ophthalmic history of the affected eye included the eye’s laterality and the previous history of PDR, RVO, retinal arterial macroaneurysm (RAM), nAMD, and retinal tear with or without combined RD. The status of the fellow eye was assessed and included in this study. The fellow eye data involved the current presence or history of several retinal diseases, including PDR, RVO, RAM, nAMD, retinal tear, and history of PRP. The categorical variables, including systemic diseases, ophthalmic history, and the presence of retinal disease in the unaffected eye, were dichotomized in binary form according to their presence or absence. Sex was categorized as male or female.

The etiology of FOVH for each eye was determined by consensus between two retina specialists (S.K. and B.-J.C.) after a careful review of the intraoperative and postoperative findings. When two causal conditions coexisted, the most likely etiology was designated. The etiologies of FOVH were classified into six categories based on their similar treatment strategies and pathophysiology. The six categories were as follows: PDR, RVO or rupture of RAM (RVO/RAM), nAMD, retinal tear with or without RD, Terson syndrome, and other causes. RVO and the rupture of RAM were grouped together because they have common risk factors and share a similar non-surgical treatment plan in most cases [9,10]. The six classes described above were used as the target outcome for the ML algorithms to make predictions.

### 2.3. Machine Learning

Four ML algorithms were employed to build predictive models: logistic regression with the least absolute shrinkage and selection operator (LASSO), decision tree, artificial neural network (ANN), and extreme gradient boosting (XG-Boost) [11]. We selected LASSO logistic regression as a widely used representative of conventional algorithms and included XG-Boost as it has demonstrated superior performance among tree-based algorithms. ANN was chosen as a representative of non-tree-based functional methods, given its capability to learn complex patterns, including nonlinear ones. The decision tree algorithm was incorporated to provide clinicians with a descriptive and interpretable guideline. To initiate the ML process, the selection of predictive factors for the etiology of FOVH was carried out using recursive feature elimination (RFE) with cross-validation, following a methodology employed in our previous study, which is described elsewhere [12]. Briefly, RFE is a greedy selection algorithm that creates an optimal feature subset based on the backward elimination method [13]. RFE was chosen because it allows for a significantly exhaustive search of feature combinations and it is more likely to enhance the model’s predictive performance, as well as it is also compatible with most ML algorithms provided by scikit-learn, enabling consistency in the feature selection approach across algorithms. Utilizing the extracted features, the best prediction models were investigated and determined for each ML algorithm. The best hyperparameters of ML models were selected by a grid search method.

Model training and evaluation were conducted using seven-fold cross-validation to maximize the size of the training set while preserving a valid test set size. The dataset was divided into seven subsets of similar size, with each subset utilized as the test set once, while the remaining six subsets were combined as the training set [14]. The model was trained and evaluated in each iteration, and the performance metrics were averaged to assess the overall model performance [14]. To provide references comparing ML algorithms, two majority voting strategies were independently employed. The first strategy (majority voting 1) involved voting for PDR as the etiology of FOVH in all cases. The second strategy (majority voting 2) entailed voting for PDR in patients with DM and for RVO/RAM in patients without DM.

To create confusion matrices, a common test dataset was separated for the four ML algorithms. The heatmap was normalized based on the row percentages of each cell. The best hyperparameters, determined by grid search, were used for training each model with the training dataset. Subsequently, the trained ML models were employed to create the confusion matrices based on the test dataset. Feature selection and model construction were executed on the scikit-learn 0.23.1 platform in Python 3.8 (Python Software Foundation, Wilmington, DE, USA). A hardware system comprising an NVIDIA GeForce RTX 2080ti graphics processing unit (NVIDIA Corporation, Santa Clara, CA, USA) was used and each run across all algorithms was completed within 10 min during both the feature selection and model training processes.

### 2.4. Statistical Analyses

Mean accuracy was utilized to compare ML models. Continuous variables were presented as mean ± standard deviation, while categorical variables were expressed as numbers and percentages. The Fisher’s exact test was used to assess differences in categorical variables among the six etiology groups. The Kruskal–Wallis test was employed to compare continuous variables among the etiology groups, and pairwise comparisons were conducted using the Dunn–Bonferroni post hoc method. Statistical significance was set at *p* < 0.05. All analyses were performed using IBM Statistical Package for the Social Sciences software version 26 (IBM Corporation, Armonk, NY, USA).

## 3. Results

### 3.1. Clinical Characteristics of Patients

**Figure 2:**
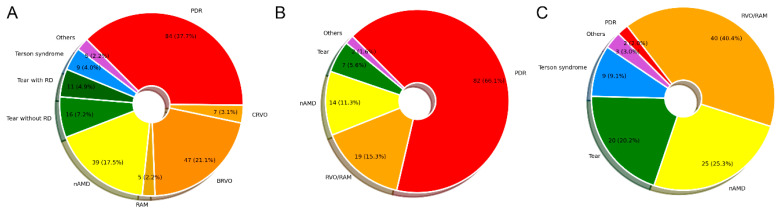
Distribution of fundus-obscuring vitreous hemorrhage etiologies among all patients (**A**), patients with diabetes mellitus (**B**), and patients without diabetes mellitus (**C**). BRVO, branch retinal vein occlusion; CRVO, central retinal vein occlusion; nAMD, neovascular age-related macular degeneration; PDR, proliferative diabetic retinopathy; RAM, retinal arterial microaneurysm; RD, retinal detachment; and RVO, retinal vein occlusion.

A total of 223 eyes of 204 patients were included in the dataset. Figure 2 shows the distribution of VH etiologies of patients. The major etiologies of FOVH were PDR (37.7%), followed by RVO/RAM (26.4%) and nAMD (17.5%). Among patients without underlying DM, the most frequent etiology of FOVH was RVO/RAM (40.4%). Two patients who had not been previously diagnosed with DM were found to have PDR after postoperative DM confirmation.

The clinical characteristics of 204 patients are presented in Table 1. Among them, 125 (56.1%) were male, and the mean age was 64.0 ± 12.8 years, which showed significant differences among groups (*p* < 0.001). The nAMD group had the highest mean age, while the Terson syndrome group had the lowest. The prevalence of underlying systemic diseases, including DM, HTN, CKD, and SAH, exhibited statistically significant differences among the groups. Among all groups, the RVO/RAM group showed the highest proportion of patients with HTN (72.9%). All patients with Terson syndrome had a history of SAH. The other group included two eyes with retinal vasculitis, two eyes with leukemic retinopathy, etc.

### 3.2. Prediction Performance of ML Models

The mean accuracies of the ML models, which served as the primary outcomes of this study, yielded the following results: the ANN achieved a mean accuracy of 76.2 ± 7.3%, followed by the XG-Boost with 74.5 ± 3.8%, the LASSO logistic regression with 74.4 ± 4.2%, and the decision tree with 68.5 ± 5.0%. The accuracy of majority voting strategy 1 was 37.7%, and that of majority voting strategy 2 was 54.7%. Notably, the accuracy of the ANN, which exhibited the best predictive performance, surpassed that of majority voting strategy 2 by 21.5%.

**Figure 3:**
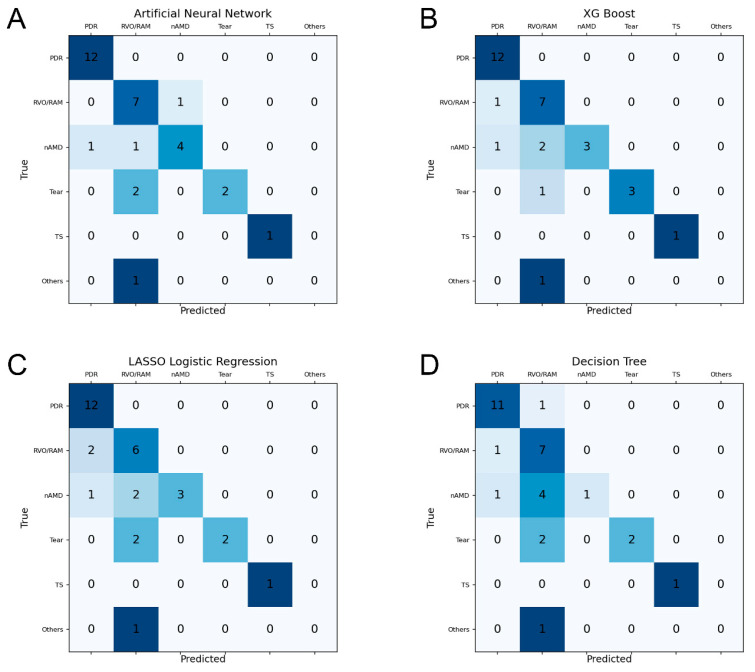
Confusion matrix heatmaps of four machine learning algorithms. (**A**) Multi-layer perceptron artificial neural network, (**B**) XG-Boost, (**C**) LASSO logistic regression, and (**D**) decision tree. LASSO, least absolute shrinkage, and selection operator; nAMD, neovascular age-related macular degeneration; PDR, proliferative diabetic retinopathy; RAM, retinal arterial microaneurysm; RVO, retinal vein occlusion; Tear, retinal tear; TS, Terson syndrome; and XG-Boost, extreme gradient boosting.

As shown in Figure 3, the heatmaps for the confusion matrices of four ML algorithms demonstrated that all algorithms achieved a high level of accuracy in predicting PDR. The RVO/RAM group was accurately predicted at a mean accuracy level of 84.4% by the four ML models. Although nAMD showed a tendency to be misclassified as RVO/RAM in algorithms except for the ANN, the ANN demonstrated the best performance in predicting nAMD by accurately predicting four out of six eyes (66.7%). Similar to nAMD, retinal tears were mis-predicted as RVO/RAM, and the mean accuracy of the four ML algorithms was 56.3%. However, XG-Boost achieved the best performance in predicting retinal tears among the four ML models by accurately predicting three out of four eyes (75.0%).

### 3.3. Common Predictive Factors and a Decision Tree

The common predictor variables identified by the three best ML algorithms including XG-Boost, LASSO logistic regression, and ANN are suggested in the Supplementary Materials, Figure S1. These features included PDR in the fellow eye, the presence of underlying DM or SAH, and a history of retinal tear, RVO, and nAMD in the involved eye. The ANN, which demonstrated superior predictive performance for FOVH among the ML models, recognized a broader range of variables as significant features. These variables encompassed demographic data, such as patients’ age, as well as comorbidities including CVA, CAD, and CKD. On the other hand, XG-Boost, which showed the best prediction for retinal tear, uniquely identified retinal tear in the fellow eye as an important feature.

**Figure 4:**
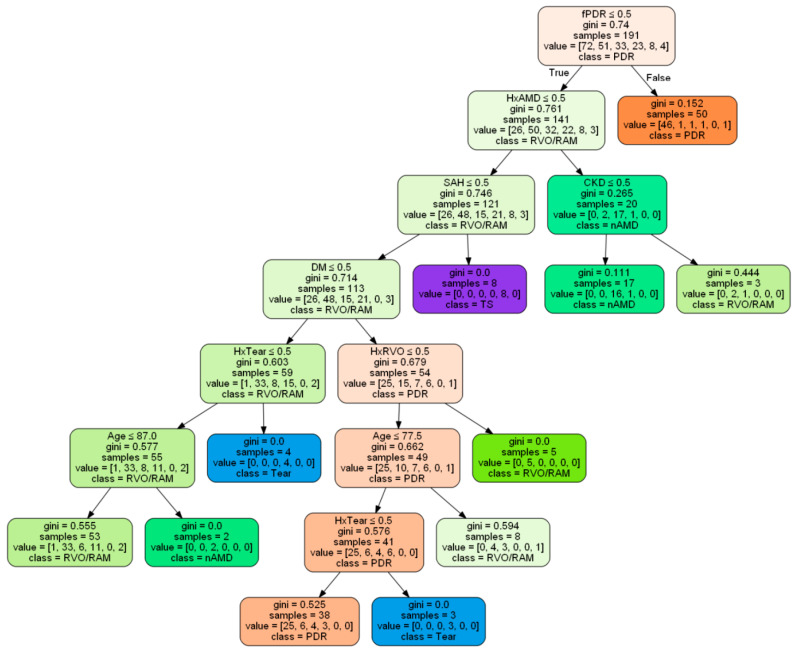
Decision tree classifying the etiology of fundus-obscuring vitreous hemorrhage. CKD, chronic kidney disease; DM, diabetes mellitus; fPDR, proliferative diabetic retinopathy in the fellow eye; HxAMD, a history of age-related macular degeneration; HxRVO, a history of retinal vein occlusion; HxTear, a history of retinal tear; nAMD, neovascular age-related macular degeneration; PDR, proliferative diabetic retinopathy; RVO/RAM, retinal vein occlusion or rupture of retinal arterial microaneurysm; SAH, subarachnoid hemorrhage; Tear, retinal tear; and TS, Terson syndrome.

The decision tree with the best performance is presented in Figure 4. The tree consisted of 10 decision nodes and 11 leaf nodes, with the first decision at the root node being the presence of PDR in the fellow eye. The highest leaf node homogeneously classified 92.0% (46 out of 50) of eyes as PDR with a Gini index of 0.152. Even though the classification had been made by the upper decision nodes, complete homogeneity was achieved in the positive leaf nodes associated with a history of SAH, RVO, or retinal tear, and in the negative leaf nodes for age ≤ 87 years old. At the final leaf nodes, there were misclassified cases: 1 out of 72 (1.4%) for PDR, 7 out of 51 (13.7%) for RVO/RAM, 15 out of 33 (45.5%) for nAMD, 15 out of 23 (65.2%) for retinal tear, zero out of eight (0%) for Terson syndrome, and four out of four (100%) for other causes.

## 4. Discussion

In the present study, ML algorithms demonstrated remarkable accuracy, reaching as high as around 75%, in predicting the unknown etiology of FOVH without visualization of the fundus status, using only preoperative clinical data. The best prediction performance was achieved by the ANN model, which exhibited an improvement of over 20% compared to a majority voting strategy (54.7%). Of note, ML models objectively suggested important predictive factors that had been vaguely considered before, such as the presence of DM, PDR in the fellow eye, and previous histories of retinal tear, nAMD, or RVO in the involved eye.

The most common cause of FOVH in this study was PDR, which is consistent with previous studies [1,2,15]. Considering that some patients without a pre-diagnosed DM had PDR in this study, it highlights the importance of ruling out PDR even in the absence of a history of DM. Among patients without DM, RVO was the main cause of spontaneous FOVH, as in previous reports [15]. The third most common etiology of FOVH was nAMD, accounting for 17.5% in the final dataset. This prevalence is higher compared to previous studies reporting only 2.0-4.3% of nAMD as a cause of VH [3,15], which might be partly attributed to the increased prevalence of nAMD over the last three decades [16]. The proportion of retinal tear (8.1%) was similar to the results in previous studies [1,2].

Preoperative research on predicting the etiology of FOVH has been limited to studies using ultrasonographic findings to determine the presence of RD or retinal tears. These studies reported sensitivity for diagnosing retinal tears ranging from 44% to 100% and specificity between 90.6% and 97% [17,18]. In contrast, our study utilized ML algorithms without ultrasonography, achieving up to 75% sensitivity and 100% specificity in predicting retinal tears and RD.

Meanwhile, the present study investigated the preoperative predictability of FOVH for the retinal tear and other various etiologies. Our findings achieved a meaningful efficacy for prediction and demonstrated the potential clinical utility of this approach. Specifically, our ML algorithms reached approximately 20% higher accuracy compared to the majority voting strategy. Also, this study proposed an approach guideline for diagnosing FOVH. It emphasized that key factors such as the presence of PDR in the fellow eye and a history of nAMD in the affected eye should be prioritized during clinical decision-making. We believe this significant improvement highlights the originality and value of our contribution to the field.

ML models achieved accurate predictions for the cause of FOVH in approximately three-quarters of the cases. Although this result could be considered fairly high, comparison with other studies was not possible because this was the first study in the literature. However, considering that an accuracy of 54.7% could be obtained simply by predicting the etiology of FOVH as PDR in diabetic patients and as RVO/RAM in non-diabetic patients, an accuracy of 75% might not be surprising. The 20% difference would be the result of utilizing various preoperative clinical parameters. ANN showed slightly better performance than XG-Boost or LASSO logistic regression, but it is difficult to explain the reasons for these small performance differences. The non-linear and non-tree-based operations that ANN can implement might have affected the results.

In terms of per-class accuracies, ML algorithms achieved excellent performance for PDR. The high frequency of PDR in the training dataset, which resulted in more patients having relevant features such as PDR in the fellow eye or the presence of DM, might have enabled this. Notably, the RVO/RAM group also exhibited a high per-class accuracy of 84.4% by ML models, but induced more false-positive predictions than PDR for nAMD or retinal tear. The distinct features of the PDR group described above might have mitigated the tendency to predict towards the majority. As expected, Terson syndrome, which occurs concurrently with SAH [19], was predicted very well with no false predictions.

Impressively, XG-Boost predicted three out of four eyes with a retinal tear without the use of ocular ultrasonography. Considering that the presence of retinal tear is of utmost importance due to the necessity of prompt treatment, our approach could help to determine the need for early PPV in patients with FOVH [6,17,20]. The unique inclusion of retinal tear in the fellow eye as an important feature by XG-Boost likely contributed to its best performance in predicting retinal tear. In fact, it is often recommended to administer prophylactic laser treatment to the fellow eye when there is a history of RD in one eye, as it increases the risk of developing RD in the fellow eye [21]. Similarly, early PPV might be recommended for eyes with FOVH and a history of RD in the fellow eye [6]. Therefore, when managing patients with FOVH, it would be crucial to thoroughly examine the retina of the contralateral eye, including the periphery.

To our surprise, the selected predictive factors by ML models appeared to be quite reasonable. Considering that diabetic retinopathy tends to manifest similarly in both eyes [22], it is appropriate to consider PDR in the fellow eye as a predictor for PDR as the etiology of FOVH. The presence of CKD, with its highest prevalence within the PDR group, could also be considered a predictive factor for PDR. Moreover, the inclusion of CAD and CVA as important features in the ANN model can be considered valid, supported by previous research indicating an association between these comorbidities and RVO [23,24]. The ophthalmic histories of RVO, nAMD, and retinal tear were also effectively utilized to predict the corresponding diseases, as shown in the decision tree. Therefore, in cases of VH with an unknown etiology, obtaining a detailed ophthalmic history of the involved eye would be highly beneficial in diagnosing the underlying cause. Additionally, the selected demographic data of the patient’s age could be regarded as a predictive factor for nAMD, considering the oldest age in the nAMD group. Expectably, SAH could be used to predict Terson syndrome [19].

It would be noteworthy that the decision tree model selected the presence of PDR in the fellow eye as the first determinant in the root node. Considering that PDR was the most common underlying cause, it is likely that the algorithm prioritized the possibility of PDR and chose the more specific feature of PDR in the fellow eye over the presence of DM. As a result, this decision allowed the tree to effectively classify PDR homogeneously at the first leaf node. Therefore, a thorough fundus examination of the fellow eye would also be a very critical and sensitive method to exclude PDR as the cause of FOVH. The eyes having a history of retinal tear, but not a history of nAMD or SAH, in non-diabetic patients should be suspected of underlying retinal tear. On the other hand, the parameter of age exceeding 87 years, as indicated by the decision tree, can be a useful predictor for identifying nAMD as the etiology of FOVH.

Traditionally, conservative management for VH has involved observing patients for several weeks, allowing for the spontaneous absorption of the hemorrhage [1]. Recent studies have suggested the potential benefits of early PPV in preventing severe vision loss, but the risk of surgical complications still exists [20,25]. Our findings could provide valuable insights into the determination of the treatment strategy of FOVH. In cases where PDR or RVO/RAM is strongly anticipated and ultrasonography does not indicate a fibrovascular membrane, anti-vascular endothelial growth factor therapy could be considered as the initial treatment instead of early PPV, despite the associated risk of tractional RD [26,27]. Treatment for severe subretinal hemorrhage in nAMD, which can cause FOVH, is still controversial and would require careful judgment by a surgeon [28]. If Terson syndrome is suspected as the underlying cause of FOVH, early PPV might be unnecessary because VH resulting from Terson syndrome primarily resolves spontaneously, and there is no significant difference in visual outcomes between early PPV and delayed PPV [29].

Our study also had several limitations. Due to the retrospective nature of this study, it would be possible that patients who were judged in need of urgent surgery by the doctors were more likely to be included, which might have induced a selection bias. Secondly, the number of data may not have been large enough to sufficiently improve the performance of the ML model. Although we attempted to address this limitation by adopting XG-Boost, a boosting algorithm, and by implementing a 7-fold cross-validation approach, applying methods to augment the dataset size could have been beneficial. Since our study classified RVO and RAM together, we could not analyze the prediction of RAM separately. Also, the feature selection method used in this study, RFE, has a limitation in that it does not accurately analyze interactions between the selected variables. Nevertheless, the current study is important because it represents the first attempt to predict the etiology of FOVH using ML algorithms, and the results demonstrated commendable accuracy. Additionally, our study could be valuable in providing not only ophthalmologists but also general physicians with a rationale for making diagnoses and treatment plans for spontaneous FOVH. Conducting further research with larger samples and an independent external validation dataset derived from real-world data would facilitate the establishment of a diagnostic flow chart and treatment guidelines for FOVH in the future.

## 5. Conclusions

In conclusion, we have identified several key risk factors associated with the underlying cause of FOVH and demonstrated that ML algorithms could predict the etiology with considerable accuracy using only preoperative information. These findings highlight the importance of comprehensive medical and ophthalmological histories and thorough fundus examination of the fellow eye in the management of FOVH. The integration of ML-based predictive tools into clinical practice has the potential to enhance diagnostic accuracy and guide therapeutic decision-making for patients with FOVH.

## Figures and Tables

**Table 1:** Clinical characteristics of the patients with fundus-obscuring vitreous hemorrhage.

	All Patients (*n* = 204)	PDR (*n* = 72)	RVO/RAM (*n* = 57)	nAMD (*n* = 38)	Tear (*n* = 26)	Terson Syndrome (*n* = 6)	Others (*n* = 5)	*p* Value
No. of eyes	223	84	59	39	27	9	5	
Age, year	64.0 ± 12.8	58.7 ± 12.3	68.6 ± 11.2	73.1 ± 11.7	61.7 ± 7.6	50.0 ± 7.3	65.4 ± 13.6	<0.001 ^a^
Male	125 (56.1)	57 (67.9)	25 (42.4)	22 (56.4)	16 (59.3)	3 (33.3)	2 (40.0)	0.032 ^b^
Right eye	114 (51.1)	43 (51.2)	29 (49.2)	21 (53.8)	13 (48.1)	5 (55.6)	3 (60.0)	0.993 ^b^
Systemic disease								
DM	124 (55.6)	82 (97.6)	19 (32.2)	14 (35.9)	7 (25.9)	0 (0)	2 (40.3)	<0.001 ^b^
HTN	131 (58.7)	47 (56.0)	43 (72.9)	23 (59.0)	16 (59.3)	0 (0)	2 (40.0)	0.001 ^b^
CKD	37 (16.6)	22 (26.2)	8 (13.6)	5 (12.8)	1 (3.7)	0 (0)	1 (20.0)	0.044 ^b^
CVA	15 (6.7)	7 (8.3)	1 (1.7)	5 (12.8)	1 (3.7)	0 (0)	1 (20.0)	0.142 ^b^
CAD	19 (8.5)	7 (8.3)	8 (13.6)	2 (5.1)	1 (3.7)	0 (0)	1 (20.0)	0.430 ^b^
SAH	9 (4.0)	0 (0)	0 (0)	0 (0)	0 (0)	9 (100.0)	0 (0)	<0.001 ^b^

Values are presented as mean ± standard deviation for continuous variables or *n* (%) for categorical variables. CAD, coronary artery disease; CKD, chronic kidney disease; CVA, cerebrovascular accident; DM, diabetes mellitus; HTN, hypertension; nAMD, neovascular age-related macular degeneration; PDR, proliferative diabetic retinopathy; RVO/RAM, retinal vein occlusion or rupture of retinal arterial macroaneurysm; SAH, subarachnoid hemorrhage; and Tear, retinal tear. ^a^ Kruskal–Wallis test; ^b^ Fisher’s exact test.

## Data Availability

The data presented in this study are available upon reasonable request to the corresponding author.

## Supplementary Materials

The following supporting information can be downloaded at https://www.mdpi.com/article/10.3390/diagnostics15030371/s1, Figure S1: Selected features by the machine learning models.

## Abbreviations

ANN	Artificial neural network
CAD	Coronary artery disease
CKD	Chronic kidney disease
CVA	Cerebrovascular accident
DM	Diabetes mellitus
FOVH	Fundus-obscuring vitreous hemorrhage
HTN	Hypertension
LASSO	Least absolute shrinkage and selection operator
ML	Machine learning
nAMD	Neovascular age-related macular degeneration
PDR	Proliferative diabetic retinopathy
PPV	Pars plana vitrectomy
PRP	Pan-retinal photocoagulation
RAM	Retinal arterial macroaneurysm
RD	Retinal detachment
RFE	Recursive feature elimination
RVO	Retinal vein occlusion
SAH	Subarachnoid hemorrhage
VH	Vitreous hemorrhage
XG-Boost	Extreme gradient boosting
