# Supplementary material for: Prediction of the Cause of Fundus-Obscuring Vitreous Hemorrhage Using Machine Learning
Source: Diagnostics (Basel). 2025 Feb 4;15(3):371. doi: 10.3390/diagnostics15030371 (PMC11817034; doi:10.3390/diagnostics15030371)
Supplement: Supplementary file 1 [file diagnostics-15-00371-s001.zip › diagnostics-3380077-supplementary.pdf]

Supplementary Figure S1. Selected features by the machine learning models.

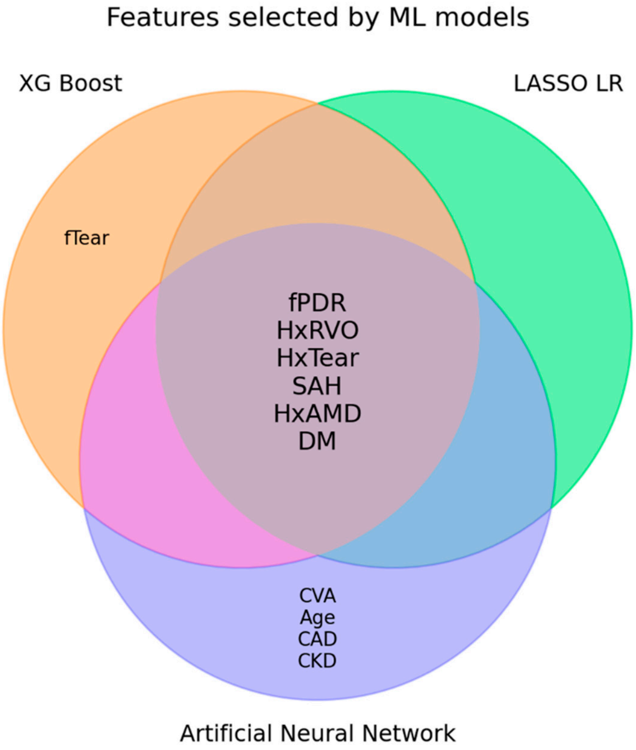

CAD, coronary artery disease; CKD, chronic kidney disease; CVA, cerebrovascular accident; DM, diabetes mellitus; fPDR, proliferative diabetic retinopathy in the fellow eye; fTear, retinal tear in the fellow eye; HxAMD, a history of age-related macular degeneration in the involved eye; HxRVO, a history of retinal vein occlusion in the involved eye; HxTear, a history of retinal tear in the involved eye; LASSO LR, least absolute shrinkage and selection operator logistic regression; ML, machine learning; SAH, subarachnoid hemorrhage; XG Boost, extreme gradient boosting.
